# Molecular Regulation of Adipogenesis and Potential Anti-Adipogenic Bioactive Molecules

**DOI:** 10.3390/ijms17010124

**Published:** 2016-01-19

**Authors:** Dorothy Moseti, Alemu Regassa, Woo-Kyun Kim

**Affiliations:** 1 Department of Animal Science, University of Manitoba, 201 Animal Science building, Winnipeg, MB R3T 2N2, Canada; mosetidorothy@gmail.com (D.M.); aregassa2005@gmail.com (W.-K.K.); 2 Department of Poultry Science, University of Georgia, 303 Poultry Science Building, Athens, GA 30602-2772, USA

**Keywords:** adipogenesis, PPARγ, C/EBPα, adipose tissue, obesity

## Abstract

Adipogenesis is the process by which precursor stem cells differentiate into lipid laden adipocytes. Adipogenesis is regulated by a complex and highly orchestrated gene expression program. In mammalian cells, the peroxisome proliferator-activated receptor γ (PPARγ), and the CCAAT/enhancer binding proteins (C/EBPs) such as C/EBPα, β and δ are considered the key early regulators of adipogenesis, while fatty acid binding protein 4 (FABP4), adiponectin, and fatty acid synthase (FAS) are responsible for the formation of mature adipocytes. Excess accumulation of lipids in the adipose tissue leads to obesity, which is associated with cardiovascular diseases, type II diabetes and other pathologies. Thus, investigating adipose tissue development and the underlying molecular mechanisms is vital to develop therapeutic agents capable of curbing the increasing incidence of obesity and related pathologies. In this review, we address the process of adipogenic differentiation, key transcription factors and proteins involved, adipogenic regulators and potential anti-adipogenic bioactive molecules.

## 1. Introduction

In the human body, excess energy intake is stored in the form of fat in the adipose tissue and during energy scarcity, this fat is released into the blood stream as fatty acids and used by other body tissues as a source of energy [1]. As such, the adipose tissue is considered an important energy store in healthy humans and serves as an essential regulator of energy balance and glucose homeostasis [2]. There are two types of adipose tissues in the human bodies; brown adipose tissue (BAT) and white adipose tissue (WAT). WAT is the most abundant and important energy storage in the form of triglycerides, while BAT is important in energy regulation through thermogenesis, which is important in heat generation in response to cold environments [3].

Obesity is mainly associated with increase in WAT commonly found under the skin and around visceral organs. Increase in size of white adipose cells in obesity leads to disruption of hormones and release of inflammatory cytokines and adipokines, which alter the normal energy homeostasis leading to a wide array of disorders such as cardiovascular diseases [4]. Secreted adipokines interfere with insulin signaling by causing insulin resistance, which in turn leads to an increase in demand for insulin production, leading to type 2 diabetes mellitus (T2DM) if production is not able to meet the demand [1].

**Figure 1:**
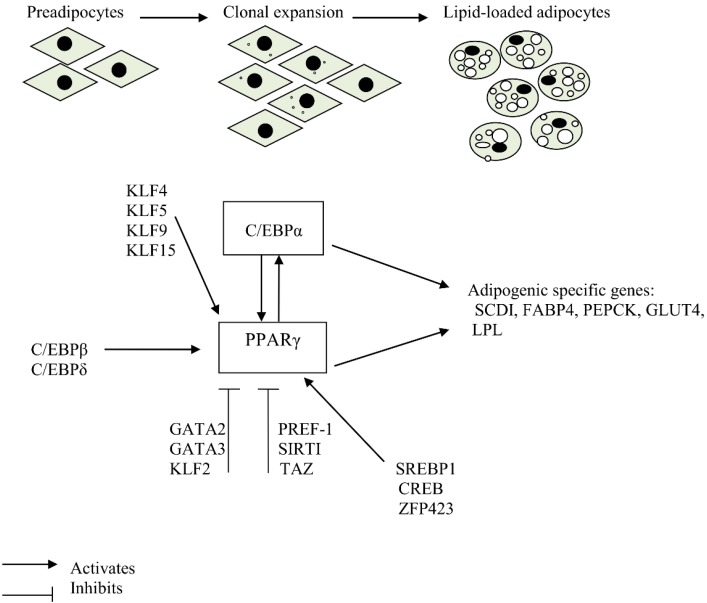
Molecular regulation of adipogenesis.

Differentiation of preadipocytes to adipocytes involves a comprehensive network including transcription factors responsible for expression of key proteins that induce mature adipocyte formation [5]. The process of adipogenesis also involves changes in cell morphology, induction of insulin sensitivity and changes in secretory capacity of cells [6]. In mammalian cells, the peroxisome proliferator-activated receptor γ (PPARγ) and CCAAT/enhancer binding protein α (C/EBPα) are the main regulators of adipogenesis and have been shown to have a broad overlap in their transcriptional targets [6]. The molecular regulation of adipogenesis is presented in Figure 1.

PPARγ is induced during differentiation of preadipocytes to adipocytes and is essential for this process [7]. Without it, precursor cells are unable to differentiate into mature adipocytes [7]. Furthermore, PPARγ is capable of promoting adipogenesis in C/EBPα-deficient cells. However, C/EBPα is not able to promote adipogenesis in PPARγ-deficient cells, demonstrating that PPARγ is the master regulator of adipogenesis [8]. Although cells deficient in C/EBPα are capable of differentiating into adipocytes, this differentiation is defective in that they accumulate less lipid droplets and do not induce expression of PPARγ, demonstrating that cross-regulation between C/EBPα and PPARγ is important for maintenance of differentiated state [8,9]. Apart from PPARγ and C/EBPα, adipocyte differentiation involves expression of several other transcription factors which interact at different stages of adipogenesis to yield mature adipocytes [10].

The expression pattern of transcripts and proteins involved in adipogenesis is in a coordinated fashion depending on the stage of adipogenesis. These transcripts and proteins regulate normal adipocyte differentiation and include glucose transporter 4 (GLUT4), lipoprotein lipase (LPL), stearyl-CoA-desaturase (SCD) and Fatty acid synthase (FAS) [11,12]. The promoters of some of the genes that are differentially expressed during the differentiation of preadipocytes to adipocytes have been shown to have binding sites for PPARγ and C/EBPα [13,14]. However, unlike mammals, PPARγ mRNA expression may not be mandatory for adipogenic induction of hen preadipocytes as it is not differentially expressed between mature adipocytes and non-differentiated control cells [15]. An understanding of the process of adipose formation and the mechanisms that govern this process is vital in the fight against the growing incidence of obesity.

## 2. Transcription Factors that Play a Crucial Role in Adipogenic Induction

### 2.1. Peroxisome Proliferator-Activated Receptors

Peroxisome Proliferator-activated Receptors (PPARs) are a nuclear hormone receptor super family of ligand-activated transcription factors, which bind to the promoter of target genes leading to increased or decreased DNA transcription upon binding of ligands, and are involved in various biological processes such as energy metabolism, cell proliferation and inflammation [16]. The PPARs consist of a non-conserved *N*-terminal domain, a highly conserved DNA binding domain (DBD), a hinge region and a *C*-terminal ligand binding domain, and anchor to their binding sites on DNA templates at the DBD leading to regulation of gene expression [17,18]. The PPAR family consists of three members, namely; α, β and γ. The name, peroxisome proliferator-activated receptor, is derived from the ability of the peroxisome proliferator-activated receptor α (PPARα) to respond to compounds that induce peroxisome proliferation [19].

PPARα mRNA is thus mostly expressed in tissues that undergo peroxisomal proliferation such as the heart, kidney and liver, where an increase in peroxisomes increases β-oxidation [20,21]. In the liver, PPARα regulates nutrient metabolism including gluconeogenesis and amino acid metabolism. It also mediates the uptake, activation and oxidation of fatty acids, synthesis of ketone bodies and apolipoproteins [16]. In addition, PPARα is highly expressed in the skeletal muscle and vascular wall [22]. Natural ligands for PPARα include polyunsaturated fatty acids such as docosahexaenoic acid, eicosapentaenoic acid, linoleic acid and linolenic acid [23].

PPARβ, on the other hand, is present in many tissues, but its functions are not very clear. However, it has been proposed to mediate fatty acid-controlled differentiation of preadipocytes [24]. PPARγ mRNA is abundantly expressed in white and brown adipose tissue, colon, cecum and macrophages and its expression increases during adipocyte differentiation [25]. PPARγ plays a dominant role in adipogenic differentiation, glucose metabolism, inflammation and other physiological processes, and is also a receptor of an important class of anti-diabetic drugs [19,26]. These drugs, known as Thiazolidinediones (TZDs), include troglitazone and are agonists/ligands of PPARγ [16]. In patients with T2DM, which is associated with lack of insulin responsiveness, activation of PPARγ by the synthetic drugs TZDs enhances insulin sensitivity leading to enhanced glucose uptake and, thus, a reduction in concentration of plasma glucose [27].

PPARγ forms a heterodimer with retinoid X receptor (RXR), enabling it to bind to DR-1 sites on target sequences [14]. Activation of PPARγ has been shown to facilitate the process of adipogenesis, leading to an increased number of small and insulin sensitive adipocytes [28]. In addition, activation of PPARγ up-regulates the adipose-derived hormone adiponectin which improves insulin sensitivity in the liver and muscle [29].

A wide range of compounds including fatty acids, prostaglandins (PGJ) and oxidized phospholipids have been proposed to act as PPAR ligands *in vitro* [16]. Some naturally occurring PPARγ agonists including 9, 10-dihydroxyoctadecenoic acid and 15-deoxy-Δ(12, 14)-PGJ(2) have been shown to act by promoting adipogenesis while inhibiting osteogenesis [30]. However, other PPARγ agonists such as 9, 10-epoxyoctadecenoic acid and the thiazolidine acetamide ligand GW0072 inhibit mesenchymal stem cells (MSC) osteogenesis but do not stimulate adipogenesis [30]. In contrast, 9-hydroxyoctadecadienoic acid stimulates adipogenesis but has no effect on osteoblast differentiation, indicating that the adipogenic and anti-osteoblastogenic effects of PPARγ are mediated by distinct pathways that are modulated by the nature of ligands involved [30].

PPARγ is expressed in two major protein isoforms: PPARγ1 and PPARγ2, which occur as a result of alternate promoter usage and splicing [31]. Both isoforms are abundantly expressed in the adipose tissue. In addition, PPARγ1 is also broadly expressed in the colon, retina and hematopoietic cells and has also been detected in low levels in the liver, spleen and heart [32,33,34]. PPARγ2 is identical to PPARγ1 except that its N-terminus contains an additional 30 amino acids. The functional differences between these two isoforms in adipocyte differentiation have been studied by blocking PPARγ2 expression in 3T3-L1 cells using artificial zinc finger repressor proteins, where cells with a 95% reduction in PPARγ2 expression failed to undergo adipogenic differentiation, but exogenous delivery of PPARγ2 into the cells restored adipogenic differentiation [35]. On the other hand, exogenous reactivation by PPARγ1 had no effect on adipogenic differentiation, suggesting that PPARγ2, not PPARγ1, plays a key role in adipogenesis [35].

PPARγ2 has been described as an adipocyte-specific nuclear hormone receptor which is capable of activating the adipocyte-specific aP2 enhancer in heterologous cells, and can be transcriptionally activated by lipids, such as polyunsaturated fatty acids [36]. *In vivo* and *in vitro* loss-of-function studies have demonstrated that PPARγ is both necessary and sufficient for adipogenic differentiation and induction of an adipose phenotype, which is marked by accumulation of lipid and expression of adipocyte differentiation markers [7,37,38]. In an experiment comparing adipogenic differentiation between PPARγ wild type and PPARγ knockout mice, it was reported that adipose tissue forms preferentially from wild type cells while PPARγ-null cells were unable to contribute to fat cell formation in the mice [37]. PPARγ knockout mice have been shown to die during embryogenesis due to interference with terminal differentiation of the trophoblast and placental insufficiency [38]. In another study, embryonic fibroblasts derived from PPARγ deficient fetuses were unable to differentiate into adipocytes in an *in vitro* model [39].

Since PPARγ plays an important role in adipogenic differentiation and is a receptor for insulin sensitizing drugs, regulation of its expression is of importance with respect to nutrition, obesity and diabetes. Tissue expression and potential for regulation of PPARγ have been studied both *in vivo* and *in vitro* [40]. In an *in vivo* study using mice, it was reported that PPARγ mRNA and protein levels are down-regulated by fasting and insulin-deficient diabetes while a diet rich in fatty acids increases adipose tissue expression of PPARγ in normal mice and induces PPARγ2 expression in the liver of obese mice [32]. Fasting for 48 hours was shown to reduce the expression of both PPARγ isoforms in subcutaneous and visceral adipose tissues of rats [33]. In an *in vitro* study, treatment of isolated human adipocytes with insulin and corticosteroids was shown to induce the expression of PPARγ mRNA [41]. In contrast, treatment of 3T3-L1 cells with Tumor necrosis factor α (TNFα), a polypeptide hormone with pleiotropic effects on cellular differentiation, down-regulated the expression of PPARγ [42].

### 2.2. CCAAT/Enhancer-Binding Proteins

The CCAAT/enhancer-binding Proteins (C/EBPs) also play an important role in adipocyte differentiation (Figure 1). They belong to a family of highly conserved basic leucine zipper transcription factors consisting of six members, where C/EBPα, -β, and –δ family members are more established in adipogenesis. In particular, C/EBPα is commonly expressed in the adipose tissue, liver, lung, adrenal gland and placenta [43,44]. C/EBPα and PPARγ are involved in a single adipogenic differentiation program, in which PPARγ plays a dominant role. C/EBPα is important in terminal differentiation of adipocytes, as absence of this factor leads to insulin resistance in *in vitro* experiments and hinders formation of WAT *in vivo*. In contrast, development of BAT is independent of C/EBPα [45,46].

C/EBPβ and C/EBPδ have been postulated to be the first transcription factors induced during induction of adipogenesis, and, therefore, play an important role in directing the differentiation process [47]. The importance of C/EBPβ and C/EBPδ has been demonstrated in loss-of-function and gain-of-function studies where embryonic fibroblasts from mice lacking these two markers were unable to differentiate in response to hormonal induction. Consequently, these cells failed to express other important adipogenic markers such as C/EBPα, PPARγ or adipocyte binding protein 2 (aP2)/Fatty acid binding protein 4 (FABP4), suggesting that *in vitro* adipocyte differentiation proceeds according to the proposed transcriptional cascade in which C/EBPs and PPAR families of transcriptional factors are activated sequentially leading to formation of mature adipocytes [48]. In contrast, *in vivo* studies show that induction of C/EBPα and PPARγ can take place without the expression of C/EBPβ and C/EBPδ. However, adipogenesis in C/EBPβ and C/EBPδ-null mice is severely impaired, suggesting that co-expression of C/EBPα and PPARγ is not sufficient for complete adipocyte differentiation in the absence of C/EBPβ and C/EBPδ [48].

## 3. Stages of Adipocyte Differentiation

Adipocyte differentiation is characterized by chronological changes in the expression of various genes that lead to the establishment of the adipocyte phenotype. These changes include the appearance of early, intermediate and late mRNA/protein markers and accumulation of triglycerides [49]. The process of adipogenesis occurs in four main stages, namely: growth arrest, mitotic clonal expansion (MCE), early differentiation and terminal differentiation [5,49]. Cultured preadipocytes undergo proliferation before entering the growth arrest stage, at which point they begin to express early markers of differentiation. Cell to cell contact may play a role in activating mechanism(s) that induce early differentiation markers [50,51].

Appropriate inducers are required for the cells to proceed to the mitotic clonal expansion stage and subsequent differentiation [52]. 3T3-L1 fibroblasts are able to differentiate into fat-laden adipocytes in a span of approximately one week upon induction using fetal bovine serum (FBS), Dexamethasone (DEX), isobutylmethylxanthin (IBMX) and Insulin [5,53]. This cocktail activates the adipogenic program in these cells, which are then directed to the different stages of adipogenesis. In particular, DEX and IBMX have been identified as direct inducers of genes responsible for the expression of C/EBPδ and C/EBPβ, respectively [54]. Insulin acts by stimulating the cells to take up glucose, which is stored in the form of triacylglycerol [55]. During the early stages of differentiation, there is a high expression of C/EBPδ and C/EBPβ in response to hormonal induction. These two transcription factors play early catalytic roles in the adipogenic differentiation pathway and diminish during late stages of differentiation, and are replaced by PPARγ and C/EBPα [44,54].

Other studies have shown that ectopic expression of C/EBPβ in NIH 3T3 fibroblasts, alone or in combination with C/EBPδ, leads to expression of PPARγ2 and eventual conversion of fibroblasts to adipocytes. However, these cells do not express C/EBPα despite accumulation of abundant lipid droplets in response to activation of PPARγ [56,57]. MCE is an important requirement for terminal differentiation. Blocking entry of 3T3-L1 cells into S phase during MCE leads to inhibition of adipogenic differentiation because it is during the MCE stage that cells express various transcription factors and regulators that lead to expression of PPARγ and C/EBPα [58].

Upon activation, PPARγ induces the expression of other target genes involved in adipogenesis. PPARγ also induces the expression of C/EBPα, which can bind on the promoter region of PPARγ, thus providing a self-regulatory loop [26]. C/EBPα induces the activation of a number of adipocyte-specific genes including stearoyl CoA desaturase-1 (SCD1), phosphoenol pyruvate carboxykinase (PEPCK), aP2 and Glucose transporter 4 (GLUT4), which contain C/EBP-binding sites in their promoter region [13,44,59]. On the other hand, target genes for PPARγ include those coding for aP2, lipoprotein lipase, acyl-CoA synthase, PEPCK, Fatty acid transport protein and adipisin, whose promoters contain regulatory elements for PPARγ [14,23,37].

Cooperative gene expression between C/EBPα and PPARγ has been demonstrated where ectopic expression of either transcription factor alone leads to expression of the other, suggesting that, at the final stage of adipogenesis, C/EBPα and PPARγ function in a cooperative manner to induce adipocyte-specific genes that establish the mature adipocyte phenotype [48]. The terminal differentiation stage is thus characterized by induction of mechanisms that are important for insulin action, lipid synthesis and transport, secretion of adipocyte specific proteins and expression of various metabolic programs that are associated with differentiated cells [5]. Maintenance of terminal differentiation is enhanced by the sustained expression of C/EBPα, which is able to *trans*-activate various adipocyte genes [13]. Furthermore, C/EBPα contains a C/EBP binding site within its proximal promoter that allows auto-activation of its own expression, which is important in enhancing continual expression of this marker [60,61].

Gene expression profiles using both microarray and qRT-PCR analyses of mRNAs obtained from adipocyte differentiation cultures have demonstrated the presence of many transcriptional proteins and receptors involved in the process of adipogenesis. Some of these receptors include liver x receptor α (LXRα) and retinoid x receptor α (RXRα), which play key roles in differentiation and maintenance of mature fat cells [62].

## 4. Positive Regulation of PPARγ Expression and Adipogenesis

### 4.1. The Kruppel-Like Factor Family

In addition to PPARγ and C/EBPα which play central roles in adipogenic differentiation, other transcription factors have been identified and shown to positively regulate adipocyte differentiation (Table 1). They include the Kruppel like factors (KLFs). The KLF zinc-finger transcription factors that are induced during adipogenesis in 3T3-L1 cell line include KLF4, KLF5, KLF9 and KLF15 [63,64,65]. KLF4 has been characterized as an early marker of adipogenic differentiation. In 3T3-L1 cells, KLF4 is expressed within the first 30 min and peaks at around 2 h after exposure to an adipogenic cocktail consisting of insulin, glucocorticoids and IBMX [63]. Further analysis shows that knockdown of KLF4 inhibits adipogenesis and down regulates the expression of C/EBPβ [63].

**Table 1:** Roles of different regulators on adipogenesis.

Regulator	Model	Effects	References
The Kruppel-like Factor Family	3T3-L1 preadipocytes	Enhanced adipogenesis	[63,64,65,66]
		↑ C/EBPα, C/EBPβ, PPARγ expression	
Sterol Regulatory Element-binding Protein 1	3T3-L1 preadipocytes	Enhanced adipogenesis	[67,68,69]
		↑ FAS, LPL, and PPARγ expression	
Cyclic AMP Response Element-binding Protein	3T3-L1 preadipocytes	Enhanced adipogenesis	[70]
		↑ PPARγ and FABP4 expression	
Zinc Finger Protein 423	NIH-3T3 fibroblasts	Enhanced adipogenesis	[71]
		↑ PPARγ expression	
	Bovine stromal vascular cells	Enhanced adipogenesis	[72]
		↑ PPARγ and C/EBPα expression	
The Kruppel-like factor 2	Mouse 3T3-L1 cell lines	Inhibited adipogenesis	[73]
		↓ PPARγ, C/EBPα, and SREBP1 expression	
GATA binding protein 2 and GATA binding protein 3	Mouse preadipocytes	Inhibited adipogenesis	[74]
		↓ PPARγ expression	
	3T3-F442A preadipocytes	Formation of protein complexes with C/EBPα and C/EBPβ	[75]
Preadipocyte factor-1	3T3-L1 preadipocytes	Inhibited adipogenesis	[76,77,78,79,80]
		↓ PPARγ, C/EBPα, FAS, SCD, and FABP4 expression	
Transcriptional-coactivator with PDZ-binding motif	C3H10T1/2 MSCs	Inhibited adipogenesis	[81,82]
	3T3-L1 preadipocytes	↓ PPARγ expression	
The histone deacetylase Sirtuin 1 (SIRT1)	C3H10T1/2 MSCs	Inhibited adipogenesis	[83]
		↓ PPARγ expression	
	3T3-L1 preadipocytes	Inhibited adipogenesis,	[84]
		↓ C/EBP-α, C/EBP-δ and FABP4 expression	

↑ increase in expression; ↓ decrease in expression.

KLF5 is induced by C/EBPδ/β during the early stages of adipogenesis in 3T3-L1 preadipocytes and is followed by expression of PPARγ2, and has been shown to bind directly to the PPARγ2 promoter and cooperate with C/EBPs to induce PPARγ2 expression [64]. This study also shows that over-expression of the dominant-negative KLF5 inhibits adipocyte differentiation while over-expression of wild type KLF5 induces adipocyte differentiation even in the absence of hormonal stimulation [64]. The expression of KLF9 is up-regulated during the middle stage of adipogenic differentiation, and inhibition of this factor by RNA interference has been shown to inhibit adipogenesis. Like KLF5, KLF9 binds directly to the PPARγ2 promoter and indirectly activates it by binding to C/EBPα [65]. The expression of KLF15 has been shown to increase during the differentiation of 3T3-L1 preadipocytes into adipocytes. Inhibition of KLF15, either by expression of a dominant negative form or by RNA interference, leads to reduced expression of PPARγ and blocked adipogenesis in 3T3-L1 preadipocytes [66]. Furthermore, ectopic expression of KLF15 in NIH 3T3 cells has been shown to induce lipid accumulation and expression of PPARγ, suggesting that KLF15 plays an important role in adipogenesis [66].

### 4.2. Sterol Regulatory Element-Binding Protein 1

The sterol regulatory element-binding protein 1 (SREBP1), also referred to as the adipocyte determination and differentiation-dependent factor 1 (ADD1), is a basic helix-loop-helix (bHLH) leucine transcription factor that is associated with adipocyte differentiation and cholesterol homeostasis [85]. SREBP1 is expressed in different types of tissues but is predominantly expressed in brown adipose tissue [67]. As a member of bHLH transcription factor family, SREBP1 has dual DNA binding specificity, in that it can bind to an E-box motif and a sterol regulatory element (SRE). Thus, when expressed in fibroblasts, SREBP1 activates transcription through both the E-box motif and SRE, providing a novel mechanism to coordinate different lipid metabolism pathways [85,86]. SREBP1 plays a role in adipocyte gene expression by regulating the expression of FAS and LPL, important genes involved in fatty acid metabolism [68].

Ectopic expression of a dominant-negative form of SREBP1 inhibits adipocyte differentiation and expression of adipocyte-specific genes in 3T3-L1 preadipocytes [68]. Furthermore, the expression of SREBP1 increases the activity of PPARγ but not that of PPARα or PPARδ, through production of endogenous ligands [10]. In another study, ectopic expression of SREBP1 in 3T3-L1 and HepG2 cells was shown to induce endogenous PPARγ mRNA levels, suggesting that SREBP1 augments adipogenic differentiation through induction of PPARγ expression [69].

### 4.3. Cyclic AMP Response Element-Binding Protein

The cyclic AMP response Element-binding protein (CREB) has been proposed to have a possible role in the control of adipogenesis. Expression of the active form of CREB in 3T3-L1 preadipocytes is sufficient to induce adipogenesis as seen by accumulation of triacylglycerols and expression of two adipocyte marker genes, PPARγ and fatty acid binding protein [70]. Alternatively, transfection of 3T3-L1 preadipocytes with a dominant-negative form of CREB blocks adipogenic differentiation [70]. Further study shows that expression of CREB is stimulated by differentiation-inducing agents such as dexamethasone, insulin and dibutyryl cAMPs [70].

### 4.4. Zinc Finger Protein 423

The zinc finger protein 423 (ZFP423) transcription factor has been identified as a regulator of preadipocyte cell determination and is abundant in preadipose compared to non-preadipose fibroblasts [71]. Ectopic expression of ZFP423 in non-adipogenic NIH 3T3 fibroblasts induces expression of PPARγ in undifferentiated cells and promotes adipogenesis once cells have been induced to differentiate, while inhibition of ZFP423 in 3T3-L1 cells blocks PPARγ expression and adipogenic differentiation [71]. Adipocyte differentiation is greatly impaired in ZFP423-deficient mouse embryos [71]. Furthermore, ZFP423 stimulates adipogenic differentiation of bovine stromal vascular cells as shown by accumulation of lipids and expression of PPARγ and C/EBPα [72]. The molecular mechanism by which ZFP423 regulates PPARγ expression is not clear, although it is proposed that it acts in part through amplification of the BMP signaling pathway [71].

## 5. Factors Negatively Regulating Adipogenesis

Bodies of scientific knowledge indicate that several factors are capable of inhibiting adipogenic differentiation in different cell lines (Table 1). The most common ones include: transcription factors, proteins, and signaling pathways. Targeting these factors would be an excellent therapeutic approach to intervene obesity and related disorders.

### 5.1. The Kruppel-Like Factor 2

The kruppel-like factor 2 (KLF2) has been identified as a transcription factor that represses adipogenesis. In mouse 3T3-L1 preadipocytes, over-expression of KLF2 inhibits the expression of PPARγ, C/EBPα and SREBP1, transcription factors that are important in adipocyte differentiation [73]. KLF2 inhibits adipogenic differentiation by binding directly to the CACCC region on the PPARγ2 proximal promoter, thereby repressing its promoter activity. On the other hand, mutation on the KLF2 binding site does not block the KLF2-mediated repression of PPARγ promoter, indicating that other mechanisms of KLF2 activity are involved [73].

### 5.2. GATA2 and GATA3 Zinc Finger Proteins

GATA2 and GATA3 are zinc-finger DNA binding proteins involved in cell development. These proteins are expressed in preadipocytes and down-regulated during the terminal differentiation process [74]. Expression of GATA2 has been shown to decrease adipocyte differentiation, while embryonic stem cells lacking GATA2 display enhanced adipogenic differentiation potential. Consequently, defective GATA2 and GATA3 expression is associated with obesity, while expression of GATA2 and GATA3 inhibits adipogenesis and traps cells at the preadipocyte stage, which could be as a result of direct suppression of PPARγ [74]. Furthermore, GATA2 and GATA3 form protein complexes with C/EBPα and C/EBPβ leading to suppression of adipocyte differentiation [75].

### 5.3. Preadipocyte Factor-1

Preadipocyte factor-1 (Pref-1) is a transmembrane protein highly expressed in preadipocytes. The inhibitory effect of Pref-1 in adipocyte differentiation has been demonstrated using various *in vitro* approaches [76,77]. Constitutive expression of Pref-1 in 3T3-L1 cells by stable transfection markedly lowers the degree of adipocyte differentiation. Pref-1 prevents lipid accumulation and expression of adipocyte transcription factors such as PPARγ and C/EBPα and other late adipocyte markers such as FAS, stearoyl-coenzyme A desaturase1 (SCD1) and ap2. Conversely, inhibiting Pref-1 expression by transfection of antisense RNA greatly enhances adipogenesis, showing that Pref-1 expression inhibits adipocyte differentiation and that down-regulation of Pref-1 is a necessary step in adipocyte differentiation. Similarly, results from *in vivo* studies in mice indicate the role of Pref-1 in adipogenesis. Mice lacking paternally expressed Pref-1/Dlk1 display growth retardation and accelerated adiposity [78].

Mice expressing the Pref-1/hFc transgene in adipose tissue showed a substantial decrease in total fat pad weight and reduced expression of adipocyte markers and adipocyte-secreted factors, including leptin and adiponectin [79]. Moreover, mice over-expressing Pref-1 were resistant to high-fat diet–induced obesity, as reflected by a marked reduction in adipose tissue mass [80]. However, Pref-1 over-expressing mice were severely insulin resistant [80].

### 5.4. Transcriptional-Coactivator with PDZ-Binding Motif

The transcriptional-coactivator with PDZ-binding motif (TAZ) has been shown to interact with 14-3-3 and PPXY motif-containing proteins through the WW domain [75,81]. Ectopic expression of TAZ in C3H10T1/2 mesenchymal stem cells (MSCs) promotes osteoblast lineage commitment through the activation of RUNX2-dependent genes and suppresses adipocyte differentiation via the repression of PPARγ activity. Contrary, diminished TAZ expression impairs osteoblast differentiation and enhances adipogenic differentiation [82]. Outcomes from mouse embryonic fibroblasts and MSCs have also indicated that TAZ coactivates Runx2-dependent gene transcription while repressing PPARγ-dependent adipogenic gene transcription [82]. Additionally, KR62980-mediated nuclear localization of TAZ has been implicated to suppress PPARγ activity and substantially inhibit rosiglitazone-induced adipocyte differentiation and attenuates adipogenic gene expression in 3T3-L1 preadipocytes [87].

### 5.5. The Histone Deacetylase Sirtuin 1 (SIRT1)

The histone deacetylase Sirtuin 1 (SIRT1) plays important roles in a wide variety of biological processes, including stress resistance, energy metabolism and differentiation [88]. Although the protein of SIRT1 has been reported to increase with that of C/EBPα during adipogenic differentiation of 3T3-L1 preadipocytes [89,90], activation of SIRT1 inhibits troglitazone mediated PPARγ expression and adipogenic differentiation in mouse C3H10T1/2 cells [83]. Similarly, over-expression of SIRT1 repressed the expression of C/EBP-α, C/EBP-δ and aP2 mRNA in 3T3-L1 preadipocytes [84]. In support of these notions, PPARγ activity and adipogenesis were promoted in SIRT1 adipocyte-specific knockout mice showing that SIRT1 plays a negative role during adipogenic differentiation [91].

## 6. Signaling Pathways Involved in Adipogenesis

The commitment of MSC towards an adipogenic or osteogenic lineage involves various signaling pathways. These pathways include: the β-catenin dependent Wnt signaling, Hedgehog signaling and Bone morphogenic protein (BMP) signaling pathways [92]. These signaling cascades influence the key regulators of adipogenesis, (PPARγ) and osteogenesis (Runx2). These factors are responsible for mediating the effects of cytokines that lead to osteogenic or adipogenic MSC differentiation, where over-expression of one factor inhibits the expression of the other [93,94]. In addition to these two factors, MSC differentiation is governed by sequential activation of a number of other transcription factors that function downstream of signaling pathways leading to lineage establishment [95].

### 6.1. Wnt Signaling Pathway

Wnt signaling pathway is a highly conserved signal transduction pathway that plays an important role in various biological processes such as regulation of cell proliferation and differentiation during embryonic development and tissue regeneration in adults [96]. Signal transduction takes place through either β-catenin dependent (canonical) pathway [96] or β-catenin independent (non-canonical) pathway which does not involve β-catenin or Wnt ligands [97,98]. Wnts are secreted glycoproteins that bind to frizzled transmembrane receptors which may be coupled to G proteins, and binding of wnt proteins to the receptors initiates signaling [99]. These glycoproteins act through paracrine and autocrine mechanisms to influence cell differentiation and development. In the β-catenin dependent Wnt signaling, β-catenin acts as the main transcriptional co-activator enhancing extracellular signal transduction for the activation of target genes [96,100].

Wnt signaling has been shown to inhibit adipocyte differentiation *in vitro*. Induction of Wnt signaling inhibits adipogenic differentiation of 3T3-L1 preadipoctyes by blocking gene expression that is responsible for mitotic clonal expansion, thus leading to dysregulation of the cell cycle [101] and blocking the expression of PPARγ and C/EBPα. Furthermore, the expression of Wnt10b, an activator of Wnt signaling, is elevated in preadipocytes and down regulated upon induction of differentiation [102]. In C3H10T1/2 cells, Wnt proteins capable of stabilizing β-catenin have been shown to induce the expression of the osteoblast differentiation marker alkaline phosphatase (ALP) while Wnt3a inhibits that expression of aP2 and PPARγ in the same cells [103]. Similarly, induction of Wnt signaling in 3T3-L1 cells inhibits adipogenesis, in part through the dysregulation of the cell cycle [101]. In contrast, disruption of Wnt signaling leads to adipogenic differentiation of pre-adipocytes and mesenchymal precursors of adipocytes [95]. Dysregulation of Wnt/β-catenin signaling has been linked to a number of human diseases such as cancer, birth defects, Alzheimer’s and osteoporosis [100,104].

### 6.2. BMP and TGF-β Signaling

Bone morphogenic protein (BMP) signaling has been identified as a downstream process that controls adipogenesis and osteogenesis [92]. BMPs, which are members of transforming growth factor-β (TGF-β) superfamily, are extracellular cytokines that induce ectopic chondrogenesis and osteogenesis [105]. BMPs are involved in a number of regulatory processes such as cellular differentiation, embryonic development and patterning of bone and cartilage tissues [106]. TGF-β and BMPs regulate the differentiation of various cell types, including adipocytes [107].

BMPs display varied effects on differentiation of MSCs, depending on the concentration and type of BMP, type of precursor cells and presence or absence of differentiation regulators. For example, BMP4 commits pluripotent C3H10T1/2 cells to an adipose lineage, allowing these cells to express adipocyte markers and display adipocyte characteristics [108]. BMP2 alone has little effect on adipogenesis but is able to interact with other differentiation factors such as TGF-β and insulin to stimulate adipogenesis in embryonic stem cells [109]. Furthermore, BMP2 causes a dose dependent differentiation of C3H10T1/2 cells where low concentrations favor adipocyte formation while high concentrations favor formation of chondrocytes and osteoblasts [110]. BMPs induce osteogenesis by binding to threonine/kinase receptors, enabling signal transduction to the nucleus through smad proteins. Moreover, nuclear cofactors cooperate with the smad proteins to regulate expression of target genes [111]. The TGF-β signaling cascade is expressed in cultured adipocytes and adipose tissue. However, *in vitro* studies show that TGF-β inhibits pre-adipocyte differentiation. In a study to identify the adipogenic transcription factors that are targeted by TGF-β, the adipogenic factors PPARγ, C/EBPβ and C/EBPδ were over-expressed in NIH 3T3 cells followed by blocking of adipogenesis using TGF-β. It was reported that TGF-β inhibits adipocyte differentiation by interacting with C/EBP and repressing its transcriptional activity [112,113].

### 6.3. Hedgehog Signaling Pathway

Hedgehog (Hh) signaling has emerged as an important modulator of stem cell differentiation processes, including adipogenic differentiation, and has been shown to play crucial roles in the developmental processes of both vertebrates and invertebrates [114].

Several studies have demonstrated the role of hedgehog signaling in MSC differentiation [115,116]. In human MSCs, activation by Hh signaling inhibits osteoblast differentiation as seen by the decrease in both mineralization and expression of osteoblastic differentiation genes such as Runx2, a key transcription factor that regulates early osteoblast differentiation [115]. During human adipocyte differentiation, Hh signaling pathway is down-regulated. Activation of this pathway impairs adipogenesis and lipid accumulation by reducing the expression of C/EBPα. However, inhibition of this pathway is not sufficient to trigger adipogenesis [116].

It has been shown that sonic Hh protein inhibits adipogenesis and expression of adipogenic differentiation markers, whereas inhibition of Hh signaling using cyclopamine increases adipogenic differentiation in 3T3-L1 [117]. It has also been reported that Hh signaling decreases during adipocyte differentiation in 3T3-L1 preadipocytes. However, decrease in Hh is not sufficient to trigger adipogenic differentiation. [118]. In C3H10T1/2 mouse cells, activation of Hh signaling has been shown to abolish adipogenic differentiation by inhibiting the expression of adipogenic transcription factors, CEBPα and PPARγ, and increasing commitment of these cells to an osteoblastic lineage [119]. Similarly, activation of Hh signaling pathway by 20(S)-hydroxycholesterol inhibits PPARγ expression and adipogenic differentiation of bone marrow stromal cells in mouse M2-10B4 cells [120].

## 7. Potential Anti-Adipogenic Bioactive Molecules

### 7.1. Oxysterols

Oxysterols are potential bioactive molecules that can regulate adipocyte formation and key adipogenic gene expression (Table 2). They are oxidized cholesterol metabolites which are commonly found in animal tissues and cholesterol-rich foods including eggs, dairy products and meats [121,122,123,124]. Storage time, heat treatment, UV-radiation significantly increase oxysterol contents in the food products [125,126,127,128]. The common oxysterols found in food products are 7-hydroxycholesterol, 7-ketocholesterol, cholesterol-5,6-epoxides, cholestane-triol, 20S-hydroxycholesterol, 25-hydroxycholesterol, and 27-hydroxycholesterol [121,129].

**Table 2:** Bioactive molecules potentially inhibiting adipogenic differentiation and enhancing osteogenic differentiation in animal and cell culture models.

Bioactive Molecule	Model	Effect	Source
20S-hydroxycholesterol	M2-10B4 bone marrow stromal cells	Inhibits adipogenesis	[15,120,130]
		↓ PPARγ expression	
	Hen preadipocytes	↓ C/EBPβ and FABP4 expression	
22S-hydroxycholesterol	M2-10B4 bone marrow stromal cells	Inhibits adipogenesis	[130]
		↓ FABP4 and LPL expression	
		Enhances mineralisation	
		↑ ALP, OCN expression	
22R-hydroxycholesterol	M2-10B4 bone marrow stromal cells	Inhibits adipogenesis and enhances mineralisation	[130]
		↓ FABP4 and LPL expression	
		↑ ALP, OCN expression	
34-hydroxycholesterol	M2-10B4 bone marrow stromal cells	Reduces adipogenesis and improves mineralization	[131]
		↓ PPARγ2, LPL and FABP4 expression	
		↑ OSX, ALP, BSP, and OCN expression	
49-hydroxycholesterol	M2-10B4 bone marrow stromal cells	Reduces adipogenesis and improves mineralization	[131]
		↓ PPARγ2, LPL and FABP4 expression	
		↑ OSX, ALP, BSP, and OCN expression	
(−)-Epigallochatechin	Mouse 3T3-L1 preadipocytes	↓ Triglyceride accumulation, ↓ PPARγ and C/EBPα expression	[132]
		Phosphorylation of AMPK and ACC	
		↑ LRP 5 and 6, DVL 2, and 3 expression	[133]
		↓ PPARγ, C/EBPα, FABP4, LPL, and FAS expression	[134]
		↓ PPARγ, C/EBPα, SREBP1c, aP2, LPL, and FAS expression	[135]
	Mice	↑ HSL, ATGL, CPT-1, and UCP2 expression	
		↓ Fat tissue formation	
Genistein	3T3-L1 preadipocytes	Inhibits adipogenesis and promote lipolysis	[136,137,138]
		↓ PPARγ and C/EBPα expression	
	Mice	↓ LPL expression and adipose tissue formation	[139]
	Human primary adipocytes	inhibits lipid accumulation	[140]
		↓ GPDH activity FABP, STREPB1, and FAS expression	
	Human adipose tissue-MSC	Inhibits adipogenic differentiation	[141]
		↓ PPARγ, GLUT-4, and SREBP-1c expression	
Resveratrol	3T3-L1 cells preadipocytes	Decreases lipid accumulation	[142]
		↓ C/EBPα, LPL, FAS, and SREBP-1c expression	
		Inhibits adipocyte differentiation	[143]
		↓ C/EBPβ, PPARγ, C/EBPα, and FABP4 expression	
	Mice	Reduces body weight	[144]
		↓ PPARγ and FAS expression	

↑ increase in expression; ↓ decrease in expression.

Studies have indicated that certain oxysterols are capable of inhibiting expression of key adipogenic transcripts and adipogenic differentiation in different species. For instance, treatment of mouse bone marrow stromal cells (MSCs) with 5 µM 20S-hydroxycholesterol (20S) inhibits PPARγ2 expression and adipogenic differentiation through a hedgehog (Hh)-dependent mechanism; the anti-adipogenic effects of 20S were completely reversed by cyclopamine, a specific Hh signaling inhibitor [120]. Similarly, treatment of hen preadipocytes with 20S reduces expression of key adipogenic transcripts [15]. In addition to Hh signaling, 20S, 22R, or 22S oxysterol at 10 µM concentration inhibits adipogenic differentiation of MSCs through extracellular signal-regulated kinases (ERK) [130]. 20S, 22R or 22S induced phosphorylated ERK and anti-adipogenic effects of these oxysterols are reversed by a specific ERK signaling inhibitor, PD98059 [130]. Furthermore, MSCs treated with Oxysterol 34 or 49 had reduced expression of adipogenic transcripts such as PPARγ2, LPL and aP2, and adipocyte formation induced by PPARγ2 activator, troglitazone, but enhanced expression of osteogenic differentiation markers such as Runx2, Osterix (OSX), ALP, bone sialoprotein (BSP) and OCN in a dose dependent manner with an EC50 of approximately 0.8 µM for Oxysterol 34 and 0.9 µM for Oxysterol 49 [131]. Recently, it was discovered that adipocytes contribute to the *de novo* synthesis of certain oxysterols. The cytochrome P450 sterol 27-hydroxylase (CYP27A1) located in the inner mitochondrial membrane was found in 3T3-L1 preadipocytes, and the concentration was increased during the adipogenic differentiation [145].

The role of CYP27A1 is to convert cholesterol into 27-hydroxycholesterol which is a cholesterol intermediate for bile acid synthesis [146]. The 3T3-L1 preadipocytes produce 27-hydroxycholesterol during adipogenesis. Interestingly, blocking CYP27A1 production and activity using a specific inhibitor (G1268267X or siRNA) significantly induced adipocyte formation and key adipogenic genes including PPARγ, C/EBPα and FABP4, and 27-hydroxychoesterol reduced adipogenic differentiation and key adipogenic gene expression in 3T3-L1 cells [145]. This suggests that 27-hydroxycholesterol has anti-adipogenic effects. However, the molecular mechanisms by which 27-hydroxycholesterol inhibits adipogenesis need to be further elucidated because individual oxysterols may have different modes of action to modulate fat tissue formation and adipogenic gene expression.

### 7.2. (−)-Epigallocatechin

(−)-epigallocatechin gallate (EGCG) is the major polyphenolic catechin in green tea [147]. Green tea has been recognized as a health beneficial drink to reduce body weight and fat deposition in the body [147,148]. Green tea contains several polyphenols including EGCG, (−)-epicatechin, (−)-epigallocatechin, (−)-epicatechin gallate [147]. Among these polyphenols, EGCG accounts for over 60% of green tea polyphenols [147].

EGCG in green tea has been reported to potentially reduce adipogenesis, fat tissue formation, and weight gain in various *in vitro* and *in vivo* studies [135,148]. The beneficial effects of EGCG may be due to its ability to inhibit adipogenesis and induce apoptosis, lipolysis and thermogenesis [149,150,151]. EGCG in green tea, when used at concentrations of 0.1, 0.2, 0.5, 1.5 and 10 µM, reduces triglyceride accumulation and inhibits the expression of PPARγ and C/EBPα, two adipogenic master regulators in mouse preadipocytes, in a dose dependent manner, with the highest reduction in the number of triglyceride cells observed at 10 µM concentration in mouse preadipocytes (3T3-L1) [132]. In addition, it has been suggested that inhibition of adipogenic differentiation by EGCG may be attributed to activation of adenosine monophosphate-activated protein kinase (AMPK) [133]. AMPK is a conserved serine/threonine kinase which is responsible for energy homeostasis and a novel target for obesity and its related chronic disorders [133,152,153]. EGCG has been shown to inhibit adipogenic differentiation of mouse 3T3-L1 cells by up-regulating phosphorylation of AMPK and its substrate, acetyl-CoA carboxylase [133]. Recently, it was reported that one of the anti-adipogenic mechanisms of EGCG is Wnt/β-catenin pathway [134]. EGCG has been shown to significantly up-regulate the expression of key Wnt signaling related genes, low density lipoprotein receptor-related protein (LRP) 5 and 6, disheveled (DVL) 2 and 3, while down-regulating key adipogenic genes, such as PPARγ, C/EBPα, FABP4, LPL, and FAS. However, β-catenin siRNA reversed anti-adipogenic effects of EGCG in 3T3-L1 preadipocytes [134]. In this study, treatment of 3T3-L1 cells with 100 µM EGCG almost completely inhibited intracellular lipid droplet formation. Further analysis showed that the effective concentration of EGCG for adipogenic inhibition was 20–400 µM [134].

EGCG has also been reported to reduce lipogenic enzymes and their activities and increase lipid oxidation and thermogenesis. EGCG significantly inhibited key genes for lipogenesis in 3T3-L1 preadipocytes such as aP2, FAS, LPL and adiponetin [134]. EGCG also enhanced the expression of uncoupling protein 2 (UCP2) gene in 3T3-L1 preadipocytes [154]. Since UCP2 is a key mitochondrial membrane transporter responsible for energy expenditure and thermogenesis [155], up-regulation of UCP2 by EGCG clearly suggests that the anti-obesity and anti-adipogenic effects of EGCG are, at least partly, attributed to an increase in energy expenditure and thermogenesis.

Many *in vivo* studies have been conducted to evaluate the effects of green tea EGCG on body weight, fat tissue formation and body metabolism change. A study of the effects of green tea EGCG supplementation on body weight gain, adipose tissue formation, and related gene expression in high fat diet-induced obese mice showed that 0.2% or 0.5% EGCG supplementation reduced body weight, adipose tissue weight, and plasma lipids (triglyceride, total cholesterol, LDL cholesterol and free fatty acids). The expression of key adipogenic and lipogenic genes including PPARγ, C/EBPα, SREBP1, aP2, LPL and FAS in epididymal white adipose tissue was also decreased [156]. However, the expression of key genes for lipolysis, β-oxidation and thermogenesis, such as hormone-sensitive lipase (HSL), adipose triglyceride lipase (ATGL), carnitine palmitoyltransferase-1 (CPT-1), and UCP2, was significantly up-regulated by EGCG [156]. In another study, 0.5% or 1% EGCG supplementation significantly attenuated fat tissue formation and body weight gain in mice fed high-fat diet [135]. EGCG supplementation also enhanced the expression of UCP2 in the liver and stearoyl-CoA desatuase-1 (SCD1), a key enzyme in fatty acid synthesis [157], in epididymal white adipose tissue, which confirms that green tea EGCG induces energy expenditure and inhibits fatty acid synthesis in order to attenuate obesity and adiposity [135].

### 7.3. Genistein

Genistein is a soybean-derived bioactive isoflavone which has several health benefits such as anti-obesity, anti-diabetes and obesity-related chronic disorders [136,158,159]. Many studies have shown that genistein inhibits adipogenesis and key adipogenic genes including PPARγ and C/EBPα and promotes lipolysis [136,137,138]. Treatment of differentiated 3T3-L1 cells with 100 µM Genistein has been shown to inhibit adipogenesis by enhancing the expression of C/EBP homologous protein, which blocks DNA binding and transcriptional activity of C/EBPβ, ultimately leading to inhibition of expression of both C/EBPα and PPARγ [138]. In a study to determine the anti-obesity potential of genistein, it was found that treatment of 3T3-L1 cells with genistein inhibited adipocyte formation in a dose-dependent manner through activation of AMP-activated protein kinase. Treatment of cells with 20–200 µM genistein significantly inhibited adipogenesis and also induced apoptosis in mature 3T3-L1 adipocytes [133]. Genistein has also been found to decrease LPL mRNA and adipose tissue formation in mice. Injection of mice with 80 and 200 mg/kg body weight genistein significantly reduced body fat by 23% and 37%, respectively, compared to the control group [139]. In another study involving 3T3-L1 preadipocytes, treatment of cells with 50 µM genistein was reported to decrease non esterified fatty acid (NEFA) content and inhibit lipid accumulation by improving endothelial nitric oxide synthase (eNOS), inhibiting the phosphorylation of P38mitogen activated protein kinase (P38 MAPK), inhibiting FAS and preventing Janus kinase 2 (JAK2)-mediated C/EBPα expression [136].

In a more recent study, genistein was found to inhibit lipid accumulation in primary human adipocytes in a dose-dependent manner and through inhibition of glycerol-3-phosphate dehydrogenase activity and adipocyte specific genes such as aP2, SREBP 1 and FAS. The inhibition was observed at concentrations of 6.25 µM and higher, with 50 µM concentration inhibiting lipid accumulation almost completely [140]. In yet another study, genistein, at concentrations of 20–100 µM reduced lipid droplet formation and inhibited adipogenic differentiation in human adipose tissue-derived MSCs in a dose-dependent manner, with the most inhibition observed at 50–100 µM concentration compared to the control vehicle [141]. Further study reported the adipogenic inhibition to occur via the Wnt/β-catenin signaling pathway, leading to inhibition of expression of various adipogenic markers such as PPARγ, GLUT-4 and SREBP-1 [141].

### 7.4. Resveratrol

Resveratrol is a polyphenolic compound found in certain plants such as grapes, and is beneficial for human health as it plays a role in the prevention of cardiovascular diseases, and has been found to have anti-inflammatory and anti-cancer properties [160,161]. Resveratrol has also been reported to have various pharmacological effects on adipocytes, is able modulate lipid metabolism and also inhibit oxidation of low density lipoproteins [142,160]. In a study to determine the effects of resveratrol on adipogenesis using 3T3-L1 cells, treatment of cells with 25 and 50 μM resveratrol significantly decreased lipid accumulation in the cells and also led to down-regulation of expression of several adipogenic markers including C/EBPα, LPL, FAS and SREBP-1c, suggesting the potential use of resveratrol in altering fat mass associated with obesity [142]. In another study involving 3T3-L1 adipocytes, treatment of cells with 20, 40 or 80 µM resveratrol decreased both lipid accumulation and expression of PPARγ, C/EBPα and SREBP-1c. This was reported to occur through activation of AMP-activated protein kinase (AMPK) [143]. A different study also reported an inhibition of lipid accumulation after two days of treatment of 3T3-L1 pre-adipocytes with 10, 20 and 40 µM resveratrol, leading to inhibition of protein expression of various adipogenic markers including C/EBPβ, PPARγ, C/EBPα and aP2 by decreasing matrix metalloproteinase-9 (MMP-9) activity [162].

Several *in vivo* studies have also reported the anti-adipogenic properties of resveratrol. In one study, mice fed on an atherogenic diet displayed increased body weight, but addition of 0.0125% resveratrol to the diet led to a decrease in body weight. Furthermore, gene expression analysis showed significantly lower expression of lipogenic genes including PPARγ and FAS in the diet containing resveratrol as compared to the atherogenic diet [144]. In a study to determine the effect of resveratrol on adipocyte adenosine triphosphate (ATP) content, it was reported that treatment of cells with 6.25–50 µM resveratrol reduced ATP content in rat adipocytes in a dose dependent manner, and this effect was observed even at low concentrations of resveratrol. This reduction in ATP caused by resveratrol was postulated to be as a result of inhibition of glucose transport and/or metabolism [163]. Recently, mice fed on an atherogenic diet containing 0.02% resveratrol were reported to have a decrease in plasma total cholesterol, low-density lipoprotein cholesterol, hepatic fatty acids, triglyceride contents and a lower body weight [164].

## 8. Role of Adenosine Monophosphate-Activated Protein Kinase (AMPK) in Adipogenesis

Adenosine monophosphate (AMP)-activated protein kinase (AMPK) is a serine/threonine protein kinase that is mostly expressed in eukaryotic cells [143]. AMPK has been described as a heterotrimeric enzyme consisting of a catalytic α subunit and two regulatory β and γ subunits, and is activated by phosphorylation of threonine 172 in the activation loop of the α subunit [165,166]. This activation comes about as a result of increase in the AMP:ATP ratio associated with metabolic stress due to a decrease in cellular ATP. Phosphorylated AMPK leads to activation of target molecules that in turn activate catabolic pathways such as fatty acid oxidation, while inhibiting anabolic pathways such as fatty acid synthesis and storage, making AMPK an important potential pharmacological target for the treatment of obesity and related metabolic disorders [143,165,167].

AMPK has also been reported to phosphorylate and regulate a number of enzymes *in vivo* such as hydroxymethylglutraryl-CoA reductase and acetyl-CoA carboxylase, important regulatory enzymes associated with sterol synthesis and fatty acid synthesis, respectively [166]. During low-energy situations, AMPK protects the cells by switching off these energy consuming pathways, *i.e.*, sterol synthesis and fatty acid synthesis [166]. AMPK thus plays a major role in energy homeostasis by acting as a fuel sensor in the regulation of glucose and lipid metabolism [167]. Activation of AMPK is also associated with inhibition of cell proliferation and apoptosis [168,169].

The role of AMPK in adipocyte differentiation has been reported in various studies. Activation of AMPK plays a role in the inhibition of adipogenesis in 3T3-L1 cells by various phytochemicals such as resveratrol, EGCG and genistein, which stimulate intracellular reactive oxygen species (ROS) release, leading to AMPK activation [133,143]. Activation of AMPK has also been linked to the inhibition of adipogenesis by dioxinodehydroeckol (DHE) in 3T3-L1 preadipocytes [170]. Similarly, activation of AMPK by 5-aminoimidazole-4-carboxamide ribonucleoside (AICAR) was found to inhibit differentiation of 3T3-L1 adipocytes by inhibiting early clonal expansion of preadipocytes and blocking the expression of late adipogenic markers including FAS and acetyl-CoA carboxylase, and transcription factors such as C/EBPα and PPARγ [171]. Conversely, inhibition of AMPK in human adipose- derived MSCs was found to promote lipid droplet formation and up-regulate adipocyte-specific genes, while down-regulating osteogenic specific genes including ALP, OCN and RUNX2, and inhibiting mineralized matrix deposition, an indication that AMPK favors osteogenic and inhibits adipogenic differentiation in MSCs [172]. AMPK is thought to inhibit fatty acid synthesis through phosphorylation of acetyl-CoA carboxylase and down-regulation of lipogenic enzyme gene transcription [166,173,174].

## 9. Other Bioactive Molecules

There are many other bioactive molecules regulating adipogenic differentiation and adipogenic key transcription factors. For example, ginsenosides, the major active molecules of Panax ginseng, have shown potential anti-obesity and anti-adipogenic effects [175]. Ginsenosides (25–100 µM) significantly reduced lipid accumulation and expression of key adipogenic genes (PPARγ and C/EBPα) [175]. Moreover, it has been shown that ginseng supplementation prevented high-fat diet induced hyperglycemia and obesity in mice [176]. Adipokines, which are secreted from adipose tissues, are important regulators for adipogenesis, insulin sensitivity, and obesity [177,178]. Among those adipokines, adiponectin increases insulin sensitivity, PPARα activity through PPAR coactivator-1α, and SIRT1-AMPK signaling system, resulting in fat oxidation, reduced lipid synthesis and prevention of hepatic steatosis [177,178]. BMPs are the transforming growth factor-β superfamily and are key regulators for adipogenesis [179,180]. In rodent and human adipose stem cells, BMP4 and BMP7 have been shown to promote transition of white adipocytes to brown adipocytes which metabolizes triglycerides to produce heat and increase energy expenditure through the expression of uncoupling protein 1 (UCP1) [179,180]. Thus, these secreted proteins can be potential molecules regulating adipogenesis and obesity in humans and animals.

## 10. Conclusions

Adipogenic differentiation is a complex process and involves the interplay of diverse transcription factors and several mechanisms. Over the last few years, significant effort has been made to identify the transcriptional processes involved in adipocyte differentiation. A proper understanding of this process is therefore vital in the quest for an intervention for weight gain, obesity, and associated pathologies. Several genome-wide studies using various *in vitro* models of adipocyte differentiation have been carried out in an attempt to map the proteins involved in adipocyte differentiation. However, more studies are needed in order to identify novel regulators of adipogenesis and appropriate targets for drugs to treat obesity and associated metabolic disorders.
